# Integrating RNA-sequencing and network analysis to explore the mechanism of topical Pien Tze Huang treatment on diabetic wounds

**DOI:** 10.3389/fphar.2023.1288406

**Published:** 2024-01-16

**Authors:** Guang-Zhao Cao, Liang-Liang Tian, Jing-Yi Hou, Yi Zhang, He Xu, Hong-Jun Yang, Jing-Jing Zhang

**Affiliations:** ^1^ Institute of Chinese Materia Medica, China Academy of Chinese Medical Sciences, Beijing, China; ^2^ Experimental Research Center, China Academy of Chinese Medical Sciences, Beijing, China

**Keywords:** Pien Tze Huang, diabetic wounds, Klrk1, Ifng, Tlr2, Nod2, NF-κB signaling pathway

## Abstract

**Introduction:** Diabetic ulcers have become one of the major complications of diabetes mellitus (DM) and are a leading cause of death and disabling disease. However, current therapies are not effective enough to meet clinical needs. A traditional Chinese medicine (TCM) formula, Pien Tze Huang (PZH), is known as a medicine that is used to treat diabetic ulcers.

**Methods:** In this study, PZH (0.05 g/cm^2^ and 0.15 g/cm^2^) and the positive drug-rhEGF were topically administered in a high-fat diet (HFD) and streptozotocin (STZ)-induced diabetic full-thickness incisional wounds, respectively. Wound healing was assessed by wound closure rate, two-photon microscope (SHG), staining with Hematoxylin and eosin (H&E), and Masson's trichrome (MTC). Then, RNA sequencing (RNA-seq) analysis, Enzyme-linked immunosorbent assay (ELISA), western blotting, and immunofluorescence (IF), network analysis, were performed.

**Results and discussion:** The results showed that PZH significantly accelerated wound healing, as well as enhanced the expression of collagen. RNA-seq analysis showed that PZH has functions on various biological processes, one of the key biological processes is inflammatory response. Tlr9, Klrk1, Nod2, Tlr2, and Ifng were identified as vital targets and the NF-κB signaling pathway was identified as the vital pathway. Additionally, PZH profoundly reduced the levels of Cleaved caspase-3 and promoted the expression of CD31 and TGF-β1. Mechanically, PZH significantly decreased expression of NKG2-D, NOD2, and TLR2, and further inhibited the activation of downstream NF-κB signaling pathway and inhibited expression of inflammatory factors (IFN-γ and IL-1β). Importantly, we found that several active ingredients may play a significant role in diabetic wound healing, including Notoginsenoside R1, Deoxycorticosterone, Ursolic acid, and 4-Methoxyphenol. In summary, our study sheds light on the complicated mechanisms underlying the promising anti-diabetic wounds of PZH and provides the discovery of agents treating diabetic ulcers.

## 1 Introduction

Diabetes mellitus (DM) is a chronic disease that seriously threatens human health. Among them, type II diabetes accounts for 90%–95% of cases in all diabetes (Edeh et al., 2022). DM along with its associated complications is a prevalent and serious global health problem, and diabetic ulcers are one of the most severe and common complications of DM, and most patients may require amputation, which is the leading cause of death (Fan et al., 2019; Kolahian et al., 2019). Wound healing, a multistep process that involves oxidative stress, inflammation, apoptosis, angiogenesis, and extracellular matrix (ECM) secretion, is dysregulated in diabetic ulcer patients (Yan et al., 2020; Hu et al., 2021; Casado-Diaz et al., 2022; Wang et al., 2022). These factors interact with each other to make the wound extremely difficult to heal. Current treatments for diabetic ulcers include the use of systemic antibiotics, conventional debridement, nonbiologic dressings, and negative pressure drainage (Jeon and Kim, 2018; Cazzell et al., 2019; Rodríguez-Rodríguez et al., 2022). However, most of these methods are expensive and time-consuming, impeding treatment. Therefore, the discovery of novel anti-diabetic wound therapeutic strategies is urgently and highly demanded to improve clinical outcomes.

PZH is a standard formula recorded in the Chinese Pharmacopoeia, is composed of four herbs, including *Panax notoginseng (Burkill) F.H.Chen* (Araliaceae; Notoginseng Radix et Rhizoma), *Moschus* (Cervidae; excretion of Moschus berezovskii Flerov), *Calculus Bovis* (Bovidae; the gall-stone of *Bos taurus* domesticus Gmelin), and *Snake Gall* (Colubridae; snake gall bladder), which has a wide range of pharmacological effects including anti-chronic wounds, anti-cancer, anti-hepatopathy, and anti-ischemic stroke (Chinese Pharmacopoeia Commission, Huang et al., 2019; Huang et al., 2021; Zheng et al., 2019; Chen et al., 2022; Zhang et al., 2023). In addition, the therapeutic benefits of PZH in type Ⅰ diabetic wound healing have been well-documented (Yan et al., 2020). Besides, our previous study has shown that intragastric administration of PZH could promote type Ⅱ diabetic wounds by inhibiting inflammation and promoting energy generation (Zhang et al., 2023). Therefore, we also wanted to explore whether topical treatment of PZH would be effective against diabetic wounds and its possible mechanism.

High-fat diet (HFD) and streptozotocin (STZ)-induced diabetic full-thickness incisional wounds model has been widely used in the studies of diabetic ulcers (Jackson et al., 2020; Xiao et al., 2022). Besides, RNA sequencing (RNA-seq) technology can facilitate the study of gene function at the overall level, and reveal the molecular mechanism of specific biological processes and disease occurrence (Wang et al., 2022). This approach has been widely used for studies of the mechanism of action (Zhang et al., 2020a; Zhang et al., 2021b). Therefore, in this study, we evaluated the effect of PZH against diabetic ulcers in a HFD and STZ-induced diabetic full-thickness incisional wounds model. To further explore its mechanism of action, RNA-seq, network analysis, Western blotting, immunofluorescence (IF) staining, and Enzyme-Linked Immunosorbent Assay (ELISA) were performed. Therefore, this study demonstrated the therapeutic effect of topical treatment of PZH in type II diabetic ulcers, and its mechanism was analyzed.

## 2 Material and methods

### 2.1 Quality control of PZH

The quality control of PZH has been performed in accordance with Chinese Pharmacopoeia Commission, 2020; Chinese Pharmacopoeia Commission, 2020). Briefly, take PZH powder (batch number: 2107111), pass through a 100-mesh sieve, weigh accurately 1 g, add internal standard solution (patchouli alcohol, 0.2 mg/mL) 2 mL, and then add 3 mL of anhydrous ethanol, ultrasonic treatment (power 300 W, frequency 40 kHz) for 10 min, weigh again and make up the lost weight with anhydrous ethanol after 2 h, 1 μL continuous filtrate was injected into the high performance liquid chromatograph. Gas chromatography was performed with a TRACE GC Ultra (Thermo Fisher Scientific, Dreieich, Germany). An HP-5 (5%phenyl/95%methyl-polysiloxane) capillary column (30 m, 0.32 mm ID, 0.25 μm film thickness) was used for separation. The PZH samples were detected under this condition: the temperature was initially 150°C for 30 min, rising to 250°C at 20°C/min for 15 min. Besides, the injection port temperature was 250°C and the detector was held at 300°C. The standard of patchouli alcohol and muscone (≥98% purity) were provided by Sichuan Weikeqi Biological Technology Co., Ltd. (Sichuan, China).

### 2.2 Animal model establishment and drug administration

Male Sprague-Dawley rats (105 ± 15 g) were obtained from Beijing Huafukang Biotechnology Co., Ltd [SCXK (BJ) 2019-0008]. All animal care and experimental procedures were conducted according to the protocol approved by the Institute of Basic Theory for Chinese Medicine, China Academy of Chinese Medical Sciences. The project identification code is No. IBTCMCACMS21-2109-01, and the approval date of the Ethics Committee is 8 September 2021. All rats were group-housed with free access to water and food in the vivarium, which had a 12-h light/dark cycle and a temperature-controlled environment. After 3-days acclimatization, rats were fed a high-fat diet with free access to water for 4 weeks. Following 12 h of fasting, rats were injected intraperitoneally with 35 mg/kg streptozotocin (STZ) to induce diabetes. DM was diagnosed as fasting blood glucose (FBG) levels higher than 16.7 mmol/L but lower than 33.3 mmol/L. Two 2-cm diameter circular full-thickness wounds were created on each side of the dorsal surface (back of forelimb) of rats, and different drugs to the left and right holes. The rats were divided into five groups: Control, Model, PZH-L, PZH-H, and positive drug. The control group and model group were applied with physiological saline. The PZH-L and PZH-H groups were applied with PZH, at a dose of 0.05 g/cm^2^ and 0.15 g/cm^2^. The rhEGF was used as the positive control, at a dose of 40 IU/cm^2^.

### 2.3 Wound closure rate assay and collagen growth on the edge of the wounds

At 0, 6, 10, and 14 days during the treatment, the wounds were photographed and the wound closure rate was calculated as follows: A =  (B−C)/B × 100%. Where A is the rate of wound closure reduction on day 0, day 6, day 10, and day 14 post-wounding, B is the initial area of the wound, and C represents the wound area at the time of measurement. Then, the rats were anesthetized and placed on a two-photon microscope (SHG) to observe the collagen growth on the edges of the wounds.

### 2.4 Hematoxylin and eosin (H&E) staining and Masson’s trichrome (MTC) staining

Tissue samples were fixed in 4% paraformaldehyde for 3 days at room temperature. Tissue blocks dehydrated in graded ethanol series, cleared in xylene, embedded in molten paraffin, and sectioned (6 μm) for H&E and MTC stains. H&E staining and MTC staining were consistent with our previous study (Zhang et al., 2023). Briefly, for H&E staining and Masson staining, the skin tissue slices were carried out according to the instructions of the manufacturer’s protocol.

### 2.5 RNA sequencing (RNA-seq) analysis

The skin tissue was poured into a mortar precooled and the liquid nitrogen was added to fully grind the sample into powder. Total RNA was extracted from skin tissue using the TRIzol reagent according to the manufacturer’s instructions, and the quality of RNA was evaluated by the 2100 Bioanalyzer. RNA-seq was performed in the platform of Illumina HiSeq 6000 in Novogene Bioinformatics Technology Co., Ltd. (Beijing, China). The raw data have been uploaded into the NCBI database SRA (PRJNA875293). The differentially expressed genes (DEGs) were selected according to *p* < 0.05 and |log2 (fold change)| ≥1 and through the volcano plot showing. The expression patterns were clustered by hierarchical clustering. Enrichment analyses were performed using DAVID, and the network of biological processes and DEGs was then constructed. The network of upregulated DEGs and downregulated DEGs after PZH treatment was constructed and enrichment analysis was performed, respectively. Besides, P. notoginseng is the main raw material of PZH, and notoginsenoside R1(NR1) is a major active component of P. notoginseng (Sun et al., 2018). Our previous research showed that NR1 facilitated wound healing by alleviating apoptosis and inflammation, at the same time increasing ECM remodeling and promoting angiogenesis (Cao et al., 2022). Besides, our previous research has proved that berberine (BBR) has a good therapeutic effect on diabetic ulcers. BBR promotes wound closure by decreasing ROS, oxidative stress, apoptosis, and increasing cell proliferation (Zhou et al., 2021). Importantly, the study of NR1, BBR, and PZH is consistent in the establishment of the type II diabetic ulcers model. To explore the mechanism of PZH against diabetic ulcers, we further compared and analyzed the RNA-seq data of NR1, BBR, and PZH. The RNA-seq data of NR1 and BBR were downloaded from the NCBI platform (BioProject PRJNA757790). DEGs were defined with the same conditions as PZH, and then the enrichment analyses were performed by Metascape. Finally, a network of DEGs and the unique biological process of PZH was constructed by STRING and visualized by Cytoscape 3.9.1 software.

### 2.6 Western blotting

Skin tissue was lysed with RIPA buffer, the protein concentration was measured by BCA protein assay, and equal protein amounts were added electrophoretically separated on precast 4%–20% Precast Gels (Meilun) and then transferred onto PVDF membranes. Following incubation with 5% nonfat milk for 2 h, the membranes were treated with primary antibodies overnight at 4°C. The primary antibodies were used as follows: RelB (10544S, CST, 1:1000), IκBα (ab76429, Abcam, 1:1000), and NKG2-D (sc-515599, Santa Cruz, 1:1000). After washing three times, the corresponding secondary antibody were added and incubated at room temperature for 2 h. Finally, the protein bands were visualized with a Gel Doc imaging system and analyzed with the ImageJ software.

### 2.7 Enzyme-linked immunosorbent assay (ELISA)

We evaluated the expression of transforming growth factor-β1 (TGF-β1) (E-EL-0162c, Elabscience), matrix metallopeptidase 9 (MMP9) (E-EL-R3021, Elabscience), interleukin-1β (IL-1β) (E-EL-R0012c, Elabscience), toll-like receptor 2 (TLR2) (E-EL-R0907c, Elabscience), Interferon Gamma (IFN-γ) (E-EL-R0009c, Elabscience) in rat skin tissue by ELISA. Briefly, skin tissue was homogenized by a tissue homogenizer in RIPA lysis buffer on ice. After centrifugation at 12,000 rpm for 15 min at 4°C, supernatants were collected and prepared for the ELISA experiment. The samples were incubated on microtiter plates for 1.5 h, followed by biotinylated secondary antibodies for 1 h. Next, the slides were incubated with a Horseradish Peroxidase (HRP) conjugate peroxidase working solution for 30 min. Finally, tetramethylbenzidine (TMB) substrate solution was added, and after 15 min incubation in the dark, stop solution was then added to the microtiter plates. Absorbance was measured at 450 nm using a microplate reader.

### 2.8 Immunofluorescence (IF) staining

Briefly, the sections had been repaired with the antigen repair solution, observed by using 0.5% Triton X-100 permeabilized, and blocked with 5% (w/v) bovine serum albumin (BSA). Then the sections were incubated with primary antibodies at 4°C overnight. Primary antibodies used were as follows: TGF-β1 (ab215715, Abcam, 1:200), MMP9 (ab58803, Abcam, 1:200), Platelet endothelial cell adhesion molecule-1 (CD31) (M1511-8, Huabio, 1:200), Cleaved caspase-3 (ab179517, Abcam, 1:200), NKG2-D (sc-515599, Santa Cruz, 1:200), and nucleotide-binding oligomerization domain containing 2 (NOD2) (sc-56168, Santa, 1:200). The slides were incubated with secondary antibodies for 60 min. Finally, nuclei were counterstained with 4′,6-diamidino-2-phenylindole (DAPI) (c0060, Solarbio), and the slides were visualized by fluorescence microscopy.

### 2.9 Network analysis

The chemical composition of PZH and the drug targets were obtained from the BATMAN-TCM database 2.0 (http://bionet.ncpsb.org.cn/batman-tcm/) and related research (Cao et al., 2021). The score cutoff  ≥ 80.88 and *p* adj < 0.05 were considered as indicators when screening the active compounds of PZH. Then, the drug targets related to PZH-regulated targets were obtained through the STRING database 12.0 (https://string-db.org/cgi/input.pl). Next, the network of active ingredients-drug targets-PZH-regulated targets was constructed and the active components were identified.

### 2.10 Statistical analysis

All the data have been subjected to a one-way analysis of variance (One-Way ANOVA) with the use of GraphPad Prism 9.0. Values are expressed as mean ± SD. *p* < 0.05 was considered statistically significant.

## 3 Results

### 3.1 Content determination of PZH

**FIGURE 1:**
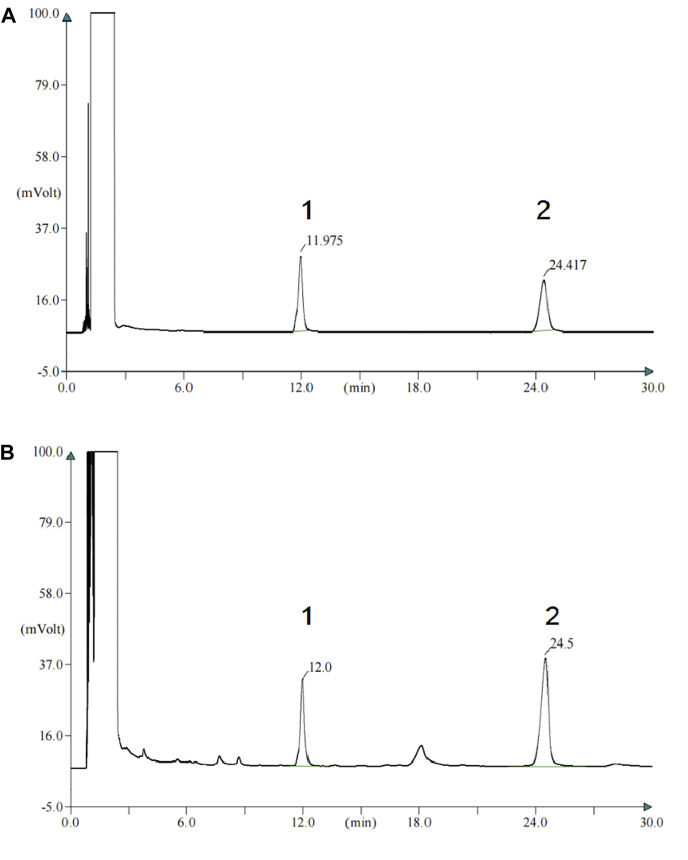
The content of muscone of PZH confirm the rules of 2020 Chinese pharmacopeia. **(A)** Gas chromatogram of the standard of patchouli alcohol and muscone. 1-patchouli alcohol; 2-muscone. **(B)** Gas chromatogram of the patchouli alcohol and muscone of PZH samples. 1-patchouli alcohol; 2-muscone.

The characteristic peaks of the patchouli alcohol and muscone of standard and PZH samples are shown in Figures 1A, B. The content of muscone was 0.435mg, which confirmed the rules of 2020 Chinese pharmacopeia.

### 3.2 Topical PZH treatment improved wound healing rate and collagen expression of diabetic rats

**FIGURE 2:**
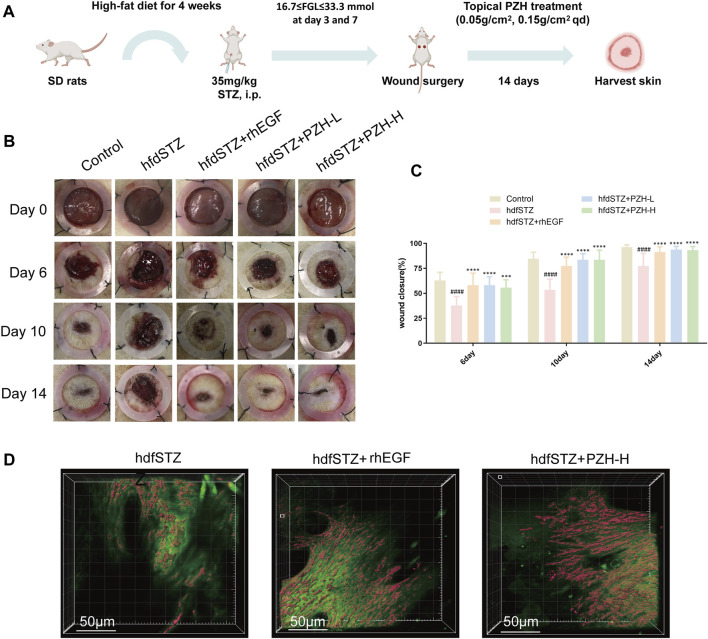
Effects of PZH on diabetic wound healing. **(A)** Overview of the model establishment and therapeutic schedule. **(B)** Images of representative wounds on day 0, day 6, day 10, and day 14 after surgery. **(C)** The wound closure rate was quantified by ImageJ software (*n* = 10). **(D)** Collagen growth on the edge of the wounds. Data were presented as the mean ± SD, ^####^ *p* < 0.0001 vs. Control, ***p < 0.001, ****p < 0.0001 vs. hfdSTZ.

The experimental flow chart is shown in Figure 2A. And the wound healing efficacy of PZH was established by investigating the wound closure rate and collagen expression. The results showed that the wound closure rate of the hdfSTZ group was significantly decreased on days 6, 10, and 14. On postoperative days 6, 10, and 14, the wound closure rate of the diabetic rats was significantly increased by PZH treatment (Figures 2B, C). As shown in Figure 2D, the SHG result showed that collagen expression around the edge of the healing wounds drastically accelerated in the hfdSTZ+PZH-H group and hfdSTZ+rhEGF group. These results indicated that topical PZH treatment enhanced wound healing in diabetic rats.

### 3.3 PZH increased ECM secretion and collagen growth in the diabetic wounds

**FIGURE 3:**
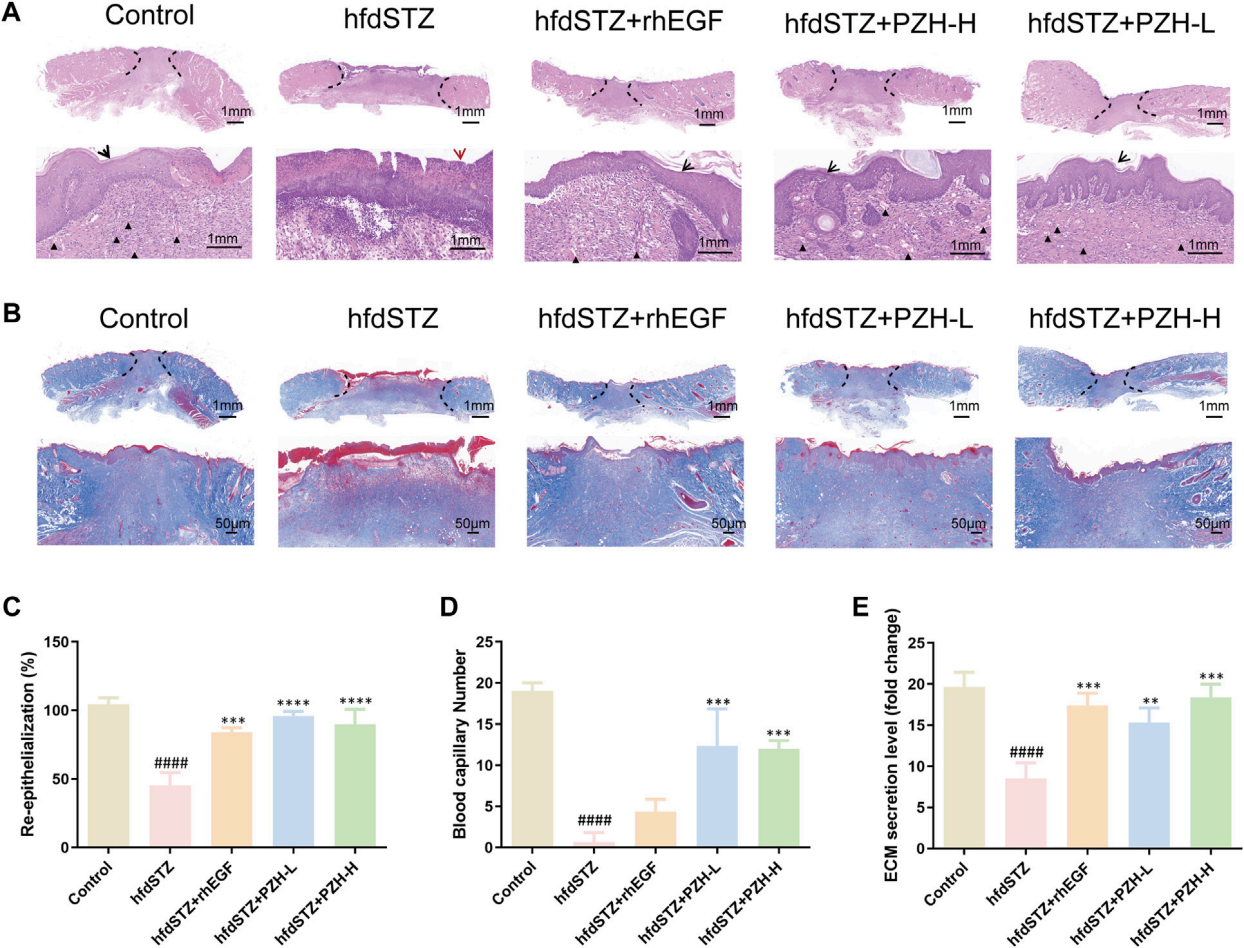
Effect of PZH treatment on ECM secretion and collagen growth in diabetic wounds. **(A)** Representative images of H&E staining. **(B)** Representative images of MTC staining. **(C)** Quantification results of re-epithelialization (*n* = 3). **(D)** Quantification results of blood capillary number (*n* = 3). **(E)** Quantification results of ECM secretion (*n* = 3). Data were presented as the mean ± SD, ^####^ *p* < 0.0001 vs. Control, **p < 0.01, ***p < 0.001, ****p < 0.0001 vs. hfdSTZ.

ECM secretion and collagen expression in response to PZH administration were evaluated by H&E staining and Masson’s trichrome staining. H&E staining results demonstrated that the re-epithelialization of the hfdSTZ group was lower in the hfdSTZ group than in the control group (red arrow), whereas the re-epithelialization was increased after treatment with PZH and rhEGF (black arrow) (Figures 3A, C). In addition, the results indicated significantly higher blood capillary number of the diabetic rats in the hfdSTZ+PZH-L group, hfdSTZ+PZH-H group, and hfdSTZ+rhEGF group than in the hdfSTZ group (black triangle) (Figures 3A, D). Furthermore, the ECM secretion in the hfdSTZ group was lower than that in the control group, but ECM secretion was significantly increased in diabetic rats treated with PZH (Figures 3A, E). As shown in Figure 3B, MTC staining showed the largest amounts of collagen expression in PZH-treated wounds compared to untreated hfdSTZ wounds.

### 3.4 The mechanism of PZH-mediated diabetic wounds was investigated via RNA-seq

**FIGURE 4:**
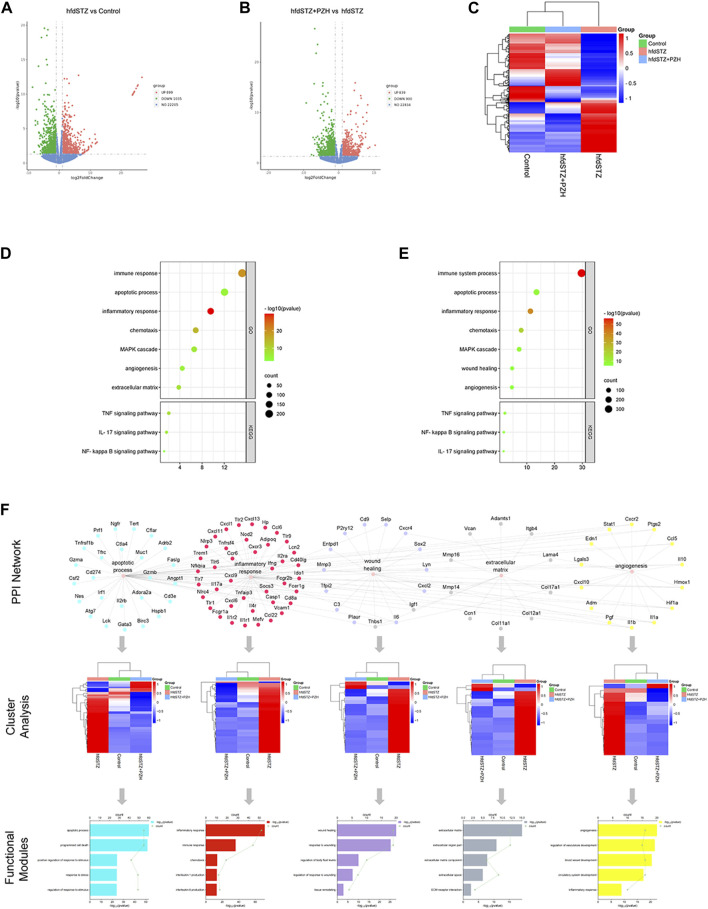
The gene expression profiling of PZH-mediated wounds in diabetic rats and the analysis of network analysis. **(A)** The volcano map of hfdSTZ vs. Control. **(B)** The volcano map of hfdSTZ+PZH vs. hfdSTZ. **(C)** The heatmap. **(D)** GO and KEGG enrichment plots of DEGs of the hfdSTZ group. **(E)** GO and KEGG enrichment plots of DEGs of the hfdSTZ+PZH group. **(F)** Network illustration of the associations among vital targets and their GO terms.

To investigate the mechanism underlying PZH against diabetic ulcers, RNA-seq was performed. RNA-seq analysis results showed that the hfdSTZ group had 899 upregulated DEGs and 1035 downregulated DEGs compared with the control group (Figure 4A). The hfdSTZ+PZH group had 839 upregulated DEGs and 900 downregulated DEGs compared with the hfdSTZ group (Figure 4B). A heatmap analysis illustrated that the hfdSTZ+PZH groups were more similar to the control group than to the STZ group (Figure 4C). As shown in Figures 4D, E, the DEGs of the hfdSTZ group were enriched in the inflammatory response, extracellular matrix, TNF signaling pathway, NF-kappa B signaling pathway, apoptotic process, angiogenesis, wound healing, etc. Whereas biological processes such as immune response, inflammatory response, apoptotic process, NF-kappa B signaling pathway, chemotaxis, PI3K-AKT signaling pathway, wound healing, angiogenesis, and extracellular matrix were significantly enriched after PZH treatment. Then, the network of DEGs enriched in biological processes associated with diabetic ulcers was constructed, such as apoptotic process, inflammatory response, wound healing, extracellular matrix, and angiogenesis (Figure 4F). The hfdSTZ group rats had different gene expression profiles compared to the control group rats, and the hfdSTZ+PZH group had more similar gene expression to the control group, respectively. The enrichment analysis showed that DEGs were not only associated with these biological processes, but also had a close connection with programmed cell death, interleukin-6 production, tissue remodeling, and blood vessel development, respectively.

### 3.5 PZH affected multiple biological processes related to diabetic wound healing

**FIGURE 5:**
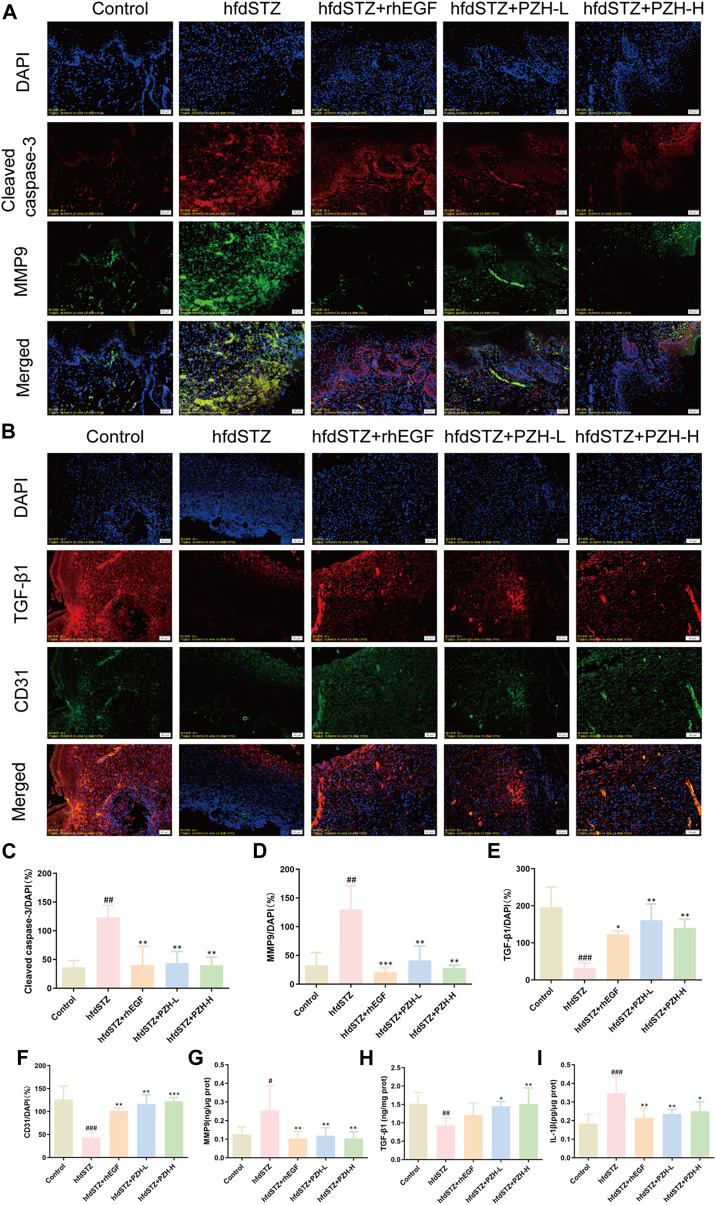
Topical PZH treatment inhibited apoptosis and inflammation and promoted ECM remodeling and angiogenesis in diabetic wounds. **(A)** IF staining for Cleaved caspase-3 (red) and MMP9 (green). Scale bar: 50 μm. **(B)** IF staining for TGF-β1 (red) and CD31 (green). Scale bar: 50 μm. **(C)** Quantification results of IF staining of Cleaved caspase-3 (*n* = 3). **(D)** Quantification results of IF staining of MMP9 (*n* = 3). **(E)** Quantification results of IF staining of TGF-β1 (*n* = 3). **(F)** Quantification results of IF staining of CD31 (*n* = 3). **(G)** MMP9 ELISA result (*n* = 6). **(H)** TGF-β1 ELISA result (*n* = 6). **(I)** IL-1β ELISA result (*n* = 6). Data were presented as the mean ± SD. Significance: ^#^ *p* < 0.05, ^##^ *p* < 0.01, ^###^ *p* < 0.001 vs. Control, *p < 0.05, **p < 0.01, ***p < 0.001 vs. hfdSTZ.

Next, the key biological processes associated with diabetic ulcers were validated. The levels of MMP9, Cleaved caspase-3, TGF-β1, and CD31 were measured. The IF staining results demonstrated that TGF-β1 and CD31 were significantly decreased while the expression levels of MMP9 and Cleaved caspase-3 were significantly increased in diabetic rats. After PZH treatment, the expression of TGF-β1 and CD31 was significantly increased and the expression of MMP9 and Cleaved caspase-3 was significantly decreased in the tissue of diabetic rats (Figures 5A–H). Besides, compared with the control group, the expression level of IL-1β in the hfdSTZ group was significantly increased. Compared with the hfdSTZ group, the level of IL-1β in the hfdSTZ+PZH-L group and hfdSTZ+PZH-H group were significantly reduced (Figure 5I). The results showed that PZH treatment inhibited apoptosis and inflammation, and increased the ECM remodeling and angiogenesis to accelerate wound healing.

### 3.6 Identification of vital targets in PZH-mediated diabetic wounds

**FIGURE 6:**
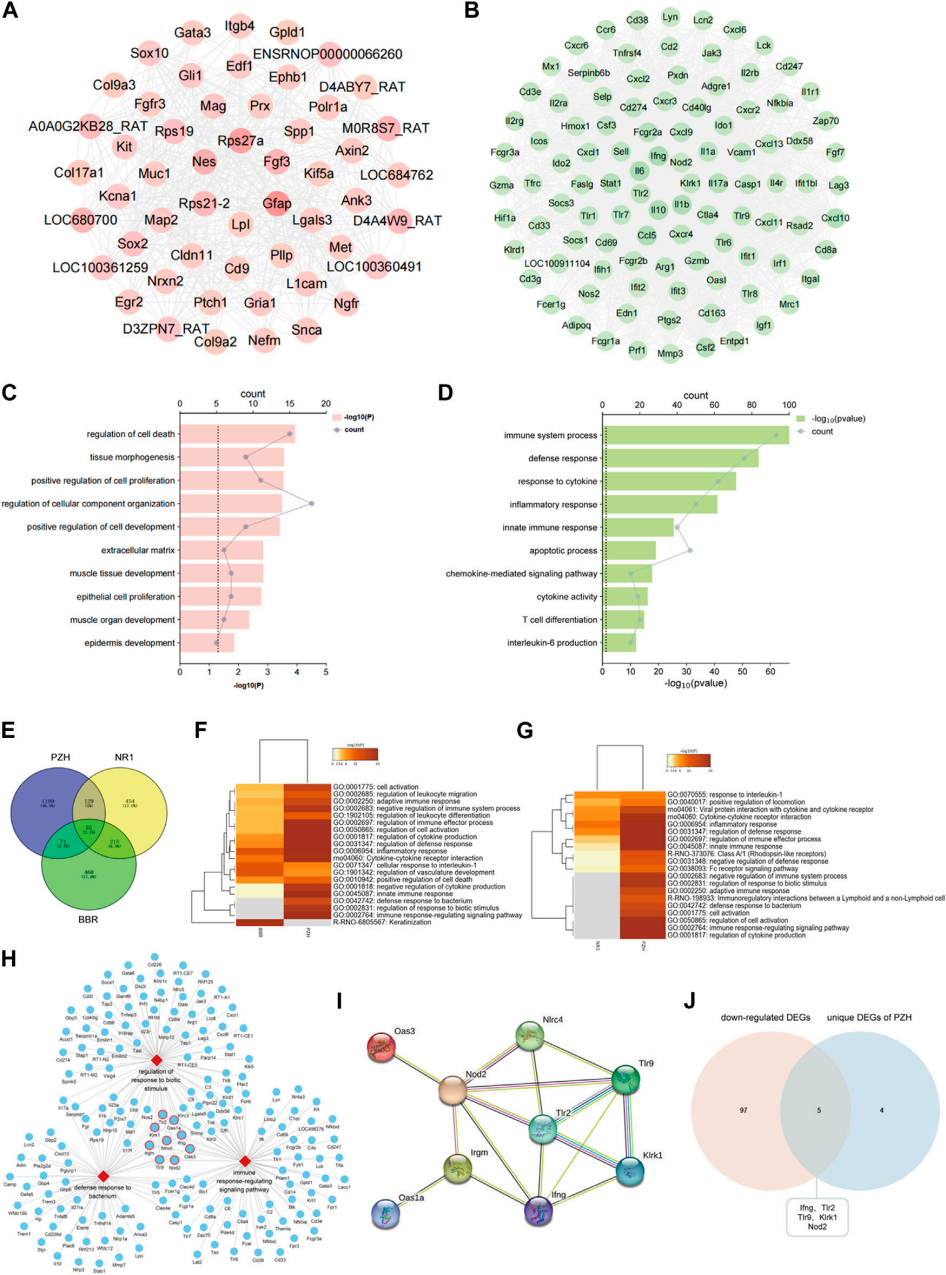
Tlr9, klrk1, Nod2, Tlr2, and Ifng were identified as vital targets. **(A)** A network of upregulated DEGs of PZH. **(B)** A network of downregulated DEGs of PZH. **(C)** The GO terms of upregulated DEGs. **(D)** The GO terms of downregulated DEGs. **(E)** Venn diagram of DEGs of PZH, BBR and NR1. **(F)** GO analysis between the PZH group and BBR group. **(G)** GO analysis between the PZH group and NR1 group. **(H)** A network of vital biological processes and potential vital targets involved in three biological processes were labeled with a red frame. **(I)** A network of potential vital targets of PZH. **(J)** Venn diagram of downregulated DEGs and unique DEGs of PZH.

To identify the vital targets of PZH against diabetic ulcers, the network of upregulated DEGs and downregulated DEGs after PZH treatment was constructed. As shown in Figures 6A, B, the targets of Rps27a, Gfap, and Nes are with the highest degree in the hfdSTZ group, and the targets of Il10, Il1b, Ifng, Il6, Tlr2, Klrk1, and Nod2 are with the highest degree in the hfdSTZ+PZH group. As shown in Figures 6C, D, the upregulated DEGs were associated with processes related to the extracellular matrix, whereas the downregulated DEGs were associated with processes related to inflammation. Through the comparative analysis of the network, it can be seen that the number of downregulated targets was more than the number of upregulated targets. Besides, the network of downregulated DEGs showed an increased probability of protein-protein interaction. Therefore, downregulated DEGs are more important than upregulated DEGs in PZH against diabetic ulcers. The results reaffirm the importance of inflammation response. We further compared the effect of PZH on diabetic wounds with that of BBR and NR1, since the same diabetic model was used. As shown in Figure 6E, 184 DEGs were found in common between the PZH group and NR1 group, 126 DEGs were found in common between the PZH group and BBR group, and 270 DEGs were found in common between the NR1 group and the BBR group. Furthermore, 55 DEGs were found in common between the three drugs. As shown in Figures 6F, G, compared with the BBR or NR1, 3 unique GO terms were found in the PZH group, such as defense response to the bacterium, immune response-regulating signaling pathway, and regulation of response to biotic stimulus. The network of these unique PZH-based biological processes network and enriched DEGs was constructed (Figure 6H). We hypothesized that targets involved in more of these three biological processes may be relatively more important because it has more functions. Thus, 9 targets including Klrk1, Nod2, Tlr2, Oas1a, Ifng, Nlrc4, Tlr9, Oas3, and Irgm were identified as potential vital targets (Figure 6I). The result of the Wayne diagram showed that 5 DEGs overlapped in the downregulated DEGs and unique DEGs in PZH (Figure 6J). Therefore, the targets of klrk1, Nod2, Tlr2, Ifng, and Tlr9 were identified as vital targets for PZH intervention in diabetic ulcers.

### 3.7 Validation of vital targets and vital pathways in PZH-mediated diabetic wounds

**FIGURE 7:**
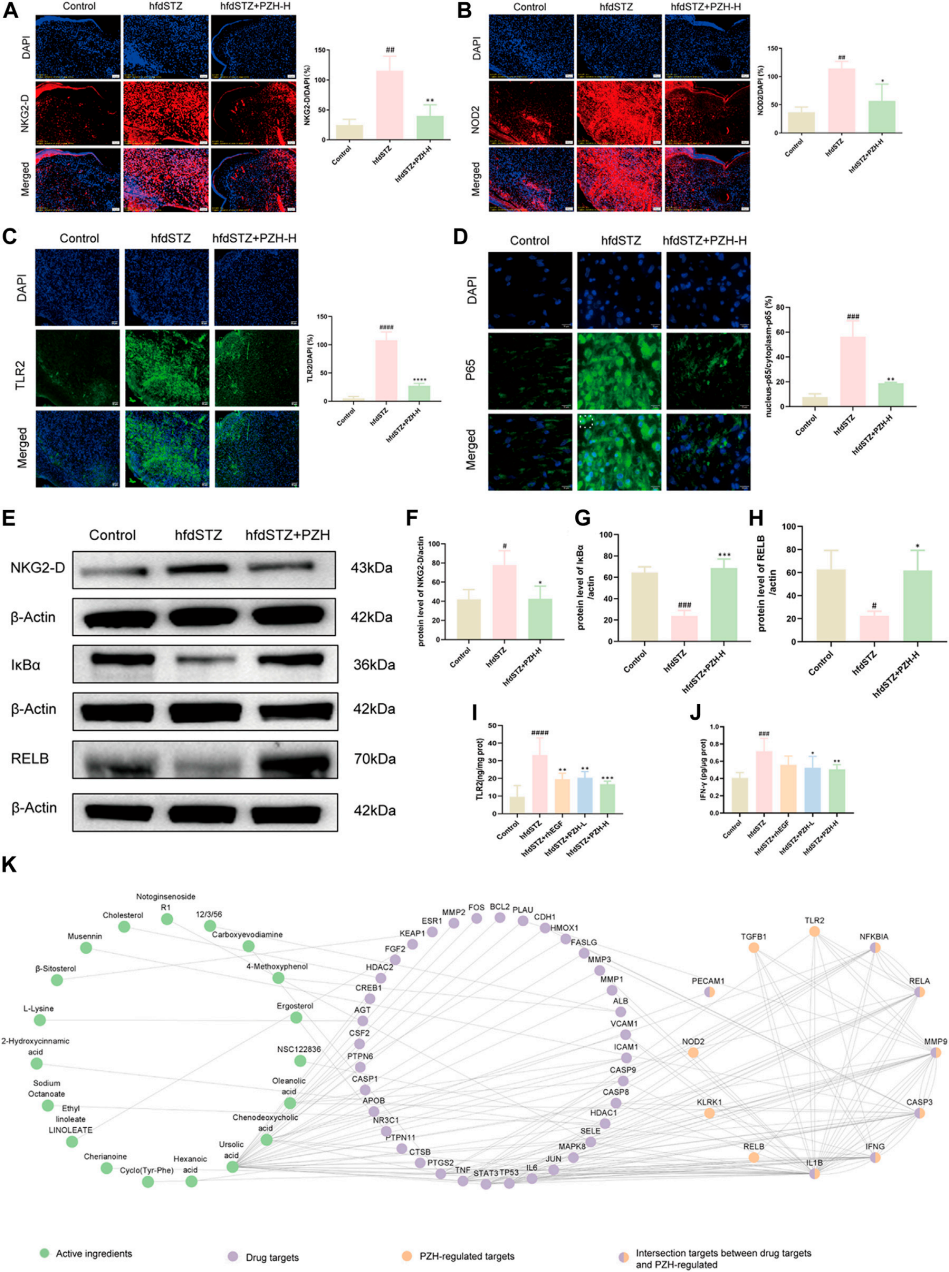
PZH significantly decreased the expression of NKG2-D, IFN-γ, NOD2, and TLR2, and inhibited NF-κB signaling pathway in diabetic wounds. **(A)** IF staining and its quantification results for NKG2-D (red). Scale bar: 50 μm (*n* = 3). **(B)** IF staining and its quantification results for NOD2 (red). Scale bar: 50 μm (*n* = 3). **(C)** IF staining and its quantification results for TLR2 (green). Scale bar: 50 μm (*n* = 3). **(D)** IF staining and its quantification results for p65 (green). Scale bar: 50 μm (*n* = 3). **(E)** Western blotting for NKG2-D, IκBα and RELB. **(F–H)** Quantification results of Western blotting of NKG2-D, IκBα and RELB (*n* = 3). **(I,J)** TLR2 and IFN-γ ELISA results (*n* = 6). **(K)** The network of active ingredients-drug targets-PZH-regulated targets. Data are presented as the mean ± SD, ^#^ *p* < 0.05, ^##^ *p* < 0.01, ^###^ *p* < 0.001, ^####^ *p* < 0.0001 vs. control, *p < 0.05, **p < 0.01, ***p < 0.001, ****p < 0.0001 vs. hfdSTZ.

**FIGURE 8:**
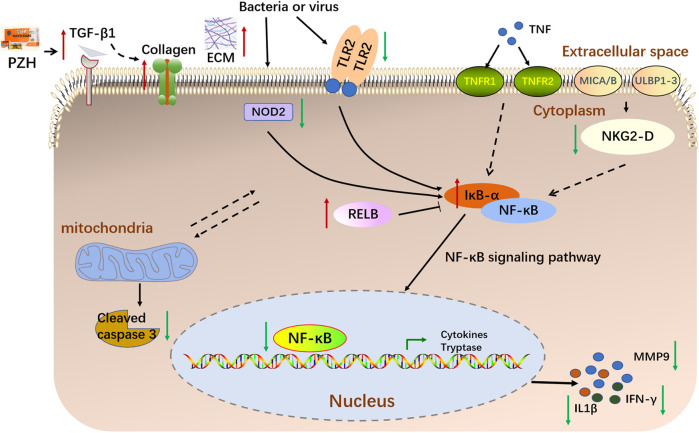
A schematic diagram illustrating the proposed mechanism underlying PZH in the treatment of diabetic ulcers.

Numerous studies demonstrate that Klrk1, Nod2, Tlr2, and Ifng are associated with various skin diseases, and excessive production of Klrk1, Nod2, Tlr2, and Ifng affects the prognosis and treatment of skin diseases (de Oliveira et al., 2016; Jiao et al., 2016; Jacquemin et al., 2020). As shown in Figures 7A–C, Figures 7E, F, and Figures 7I, J, the results showed that NKG2-D, NOD2, TLR2, and IFN-γ were markedly increased in the hfdSTZ group, whereas topical PZH treatment significantly decreased the expression of NKG2-D, NOD2, TLR2, and IFN-γ. Importantly, the increased expression of NOD2, TLR2, and NKG2-D could lead to downstream activation of NF-κB signaling pathway (Figure 8). As shown in Figures 7D, E, Figures 7G, H, RELB and IκBα were significantly decreased and nucleus-p65/cytoplasm-p65 was significantly increased in the hfdSTZ group. However, the expression of RELB and IκBα were significantly increased after PZH treatment, while the expression of nucleus-p65/cytoplasm-p65 was decreased.

To identify the active ingredients of PZH against diabetic ulcers, network analysis was performed. There were 609 drug targets of the 318 compounds of PZH were collected. And the network of active ingredient-drug targets-PZH-regulated targets was constructed, which included 19 active ingredients, 42 drug targets, and 12 PZH-regulated targets (Figure 7K). And there were 7 intersection targets between drug targets and PZH-regulated, including CASP3, IL1B, MMP9, IFNG, RELA, NFKBIA, and PECAM1. Besides, the active ingredients mainly included NR1, L-Lysine, Deoxycorticosterone, β-sitosterol, Ursolic acid, etc.

## 4 Discussion

In this study, we determined whether topical PZH could promote diabetic wound healing through an HFD and STZ injection-induced diabetic full-thickness wounds. We demonstrated that topical PZH treatment significantly improved wound closure rate and collagen growth. Given the critical role played by the PZH in the treatment of wound healing, we used the RNA-seq analysis approach to uncover the mechanism of action. RNA-seq analysis indicated that PZH facilitated the healing of diabetic ulcers by affecting critical processes such as extracellular matrix, angiogenesis, apoptosis, inflammation, etc. Importantly, inflammatory response bears a key responsibility in the treatment of PZH on diabetic ulcers. Besides, Tlr9, klrk1, Nod2, Tlr2, and Ifng were identified as vital targets and the NF-κB signaling pathway was identified as the key pathway. The further experiments demonstrated that topical PZH treatment significantly increased the expression of TGF-β1 and CD31 levels and decreased the expression of Cleaved caspase-3 and MMP9. And PZH significantly decreased the expression of NKG2-D, NOD2, and TLR2, and inhibited the activation of downstream NF-κB signaling pathways by inhibiting nucleus-p65/cytoplasm-p65 expression increasing IκBα and RELB and inhibiting expression of IFN-γ and IL-1β.

Numerous studies have indicated that difficulties in repairing wounds related to diabetic ulcers are related to the lack of ECM, excessive apoptosis, and the failure of angiogenesis (Liu et al., 2019; Ezhilarasu et al., 2020). ECM is a key element of skin repair and its dysregulation impairs wound healing in diabetes (Tian et al., 2022). ECM not only connects and supports cells and tissues, but also controls cell migration, proliferation, differentiation, and metabolism. TGF-β1 is widely recognized as playing an important role in the synthesis of ECM, inhibiting ECM degradation, and accumulating ECM by promoting the adhesion between cells and matrix (Winter et al., 2009). Furthermore, TGF-β1 might stimulate the formation of granulation tissue by increasing the ECM-related genes. Besides, TGF-β1 was proven to decrease ECM degradation by inhibiting the matrix metalloproteinases (MMPs) (Alshargabi et al., 2020). MMP9, a primary matrix-degrading enzyme, was introduced as a poor prognosis signature of tissue healing (Bradburne et al., 2013; Ciechomska et al., 2013; Abdollahi et al., 2017). And MMP9 was reported as a key inflammatory factor (Zhang et al., 2021a). A high level of MMP9 slows down the healing of diabetic ulcers by the excessive degradation of ECM (Ayuk et al., 2016). Previous studies have shown that animals deficient in MMP9 effectively promoted wound healing, indicating that MMP9 inhibits the rate of wound closure (Mohan et al., 2002). Apoptosis is a cell death pattern, which is involved in a variety of diseases (Sun and Wang, 2012). Excessive apoptosis inhibited the proliferation of epithelial cells and tissue formation of new granulation, then affected the wound healing process. Cleaved caspase-3, an executioner caspase, and its overexpression could inhibit wound healing (Tominaga et al., 2019; Cao et al., 2022). Angiogenesis is a series of complicated processes, which involves extracellular matrix proteolysis, synthesis of the new matrix, and proliferation and migration of endothelial cells (Karamysheva, 2008). It is essential for wound healing, and abnormal angiogenesis delays and impairs wound healing (Kim et al., 2020). In our study, diabetic ulcer rats showed an increase of MMP9, Cleaved caspase-3, and a decrease of TGF-β1, and CD31, which was associated with inhibited wound closure, whereas this was reversed by PZH treatment which increased ECM secretion, angiogenesis, and reduced apoptosis damage.

Importantly, diabetic patients suffer from impaired wound healing due to inflammation (Sun et al., 2020). In contrast to normal wounds, chronic wounds such as diabetic ulcers show prolonged inflammatory reactions and a correspondingly increased large number of inflammatory cytokines leading to slowed wound healing (Yang et al., 2020). IL-1β is the main regulator of tissue inflammation, the expression of IL-1β was significantly elevated in diabetic wounded skin homogenate (Younis et al., 2022). Besides, the innate immune system, especially the TLRs, takes responsibility for recognizing, responding, and regulating wounding (Murciano-Goroff et al., 2020). Chronic inflammation is frequently observed in diabetic wounds due to excessive TLR2 production (Kishibe et al., 2018). Furthermore, NOD2 plays an important role in innate immunity in the pathogenesis of diseases related to various skin diseases (Meazza et al., 2011; Jiao et al., 2016). An increased expression of NOD2 was associated with poor clinical healing outcomes in chronic wounds (Williams et al., 2018). Furthermore, the natural killer group 2D (NKG2D), a stimulatory lectin-like receptor in natural killer cells (NK) and T cells plays an important role as an activation receptor in the production of proinflammatory cytokines, including IFN-γ, on T cells. A relevant study demonstrated that NKG2D is upregulated in vitiligo skin and produces elevated levels of both IFN-γ and TNF-α (Jacquemin et al., 2020). IFN-γ, also known as IFNG, is a soluble cytokine that is produced by multiple immune cells to play an important role in both innate and adaptive immunity (Ding et al., 2022). Importantly, after binding to the bacterial products, TLR2, NOD2 and NKG2-D activate downstream NF-κB, translocate it to the nucleus, initiate the expression of inflammatory factors (Kwon et al., 2016; Kellogg et al., 2017; Zhang et al., 2020b). Furthermore, RNA-seq analysis revealed that the NF-κB signaling pathway plays a key role in attenuating diabetic wounds. Our results showed that PZH against diabetic wounds by inhibiting the expression of NKG2-D, NOD2, and TLR2, inhibiting activation of the NF-κB signaling pathway, and suppressing the release of inflammatory factors (IFN-γ and IL-1β).

Importantly, we found that there are 19 active ingredients of PZH that may play a key role in diabetic wounds, such as NR1, L-Lysine, Deoxycorticosterone, BETA-sitosterol, and Ursolic acid, etc. For example, NR1 is a novel saponin that is derived from Panax notoginseng, and our previous studies have shown the anti-diabetic ulcer effect of NR1 in rats (Cao et al., 2022). A recent study reported that 15% lysine cream can significantly improve wound healing in diabetic foot ulcer patients (Shashikumara et al., 2023). Besides, β-sitosterol treatment accelerated diabetic wound healing by promoting M2 macrophage proliferation and angiogenesis (Liu et al., 2023). Importantly, there are several unreported active ingredients that are related to diabetic ulcers, such as Deoxycorticosterone, Ursolic acid, and 4-Methoxyphenol. In summary, these active ingredients can be used as potential drugs to treat diabetic ulcers.

## 5 Conclusion

The current study systemically demonstrated that PZH significantly improved wound healing in HFD and STZ-induced diabetic rats, for example, by inhibiting apoptosis and inflammation, increasing the ECM secretion and angiogenesis. Topical treatment of PZH caused a decreased expression of NKG2-D, NOD2 and TLR2, thereby inhibiting the activation of downstream NF-κB signaling pathway, and decreasing the expression of downstream inflammatory cytokines, including IFN-γ and IL-1β. Our study not only reveals a critical mechanism underlying the anti-diabetic wounds of widely used PZH but also has important influences on the development of treatment strategies for diabetic ulcers.

## Data Availability

The original contributions presented in the study are publicly available. This data can be found here: https://www.ncbi.nlm.nih.gov/ PRJNA875293.
